# Mediterranean Fruit Fly *Ceratitis capitata* (Diptera: Tephritidae) Eggs and Larvae Responses to a Low-Oxygen/High-Nitrogen Atmosphere

**DOI:** 10.3390/insects11110802

**Published:** 2020-11-13

**Authors:** Farhan J.M. Al-Behadili, Manjree Agarwal, Wei Xu, Yonglin Ren

**Affiliations:** 1 College of Science, Health, Engineering and Education, Murdoch, WA 6150, Australia; f.al-behadili@murdoch.edu.au (F.J.M.A.-B.); m.agarwal@murdoch.edu.au (M.A.); 2 College of Agriculture, Misan University, Misan 62001, Iraq

**Keywords:** postharvest treatment, low oxygen, high nitrogen, tephritid fruit fly

## Abstract

**Simple Summary:**

Many chemicals have been removed from registration for the postharvest treatment of insect pests due to consumer/environmental safety and phytotoxicity. There is very limited operation for international trade purposes, particularly for management of Mediterranean fruit fly Ceratitis capitata (Diptera: Tephritidae) on harvested fruit. Therefore, the non-chemical method is being considered for postharvest treatment of fruit. This study explored and evaluated Medfly response to low-oxygen and high-nitrogen treatment. The results will guide the development of a novel postharvest strategy and the approach to controlling this destructive fruit fly and other pests.

**Abstract:**

The Mediterranean fruit fly, *Ceratitis capitata* (Wiedemann) (Diptera: Tephritidae), is one of the most damaging horticultural insect pests. This study used a low-oxygen/high-nitrogen bioassay to control *C. capitata*. Two low-oxygen treatments were applied (0.5% O_2_ + 99.5 N_2_ and 5% O_2_ + 95% N_2_) to *C. capitata* eggs and 1st, 2nd and 3rd instar larvae from 0 to nine days on a carrot diet at 25 °C; 70—75% RH. The pupariation, adult emergence, and sex ratios of survived flies were examined. The results demonstrate that increased mortality of all tested life stages correlated with increased exposure times at both levels of low-oxygen treatments. Complete control of eggs was achieved after eight days and nine days for larvae using 0.5% O_2_ at 25 °C; 70–75% RH. The 3rd instar was the most tolerant stage, while the egg was the most susceptible stage to the low-oxygen environment. There were no significant differences in sex ratios between emerged adults after low-oxygen and control treatments. The present work demonstrates and confirms the mortalities of *C. capitata* caused by low-oxygen treatment, which may help develop new postharvest strategies to control this destructive fruit fly pest.

## 1. Introduction

Globally, one of the most damaging horticultural insect pests is the Mediterranean fruit fly, *Ceratitis capitata* (Wiedemann) (Diptera: Tephritidae) [1]. *C. capitata* has been recorded feeding on over 300 host plants. After mating, one female adult can lay as many as 800 eggs during its lifetime [2]. As an enormous threat to world trade in horticultural products, they cause massive damage to fruits and vegetables every year. Consequently, it is a world-wide biosecurity threat [3,4].

In recent years, strict government biosecurity policies have been implemented to minimize the risks and damage *C. capitata* causes. Pre-harvest actions include sterile insect technique (SIT), insecticides, monitoring mass trapping, male annihilation and inspections, and postharvest treatments such as fumigation, low-oxygen, irradiation, heat or cold treatments have been widely applied to control fruit fly pest species. Due to public concerns on chemical residues, insect resistance and environmental pollution, non-chemical techniques have become more and more preferred in postharvest control [5,6]. Controlled atmosphere using carbon dioxide, oxygen, and/or nitrogen, together with controlled temperature and humidity, have been used for postharvest treatments against various fruit flies [7,8].

Oxygen plays a vital role in animal development [9]. A previous study showed that low-oxygen affects the developmental rate of *Drosophila melanogaster* [10]. Reducing oxygen concentration is one of the methods that has been increasingly used to control postharvest pests at room temperature [11,12]. Low-oxygen treatment to control fruit fly is one of the most promising options for the horticulture industry because low oxygen can kill insects while improving the quality of some fruits [13], for example, prevention of surface browning caused by the enzyme polyphenol oxidase [14], increasing the shelf life of plant products by reducing their respiration rates and slowing the use of the finite energy supplies that are available in any living tissue [15], and maintaining edible quality, firmness, soluble solids and acidity [16]. Furthermore, low-oxygen treatment can slow down metabolic processes, senescence, and conversion of starch, hinder fruit ripening, suppress ethanol production, inhibit certain storage disorders and decrease the growth of decay organisms [17,18]. In general, lower survival rates of insect pests can be achieved at a lower oxygen level, whenever the temperature rises above 25 °C [19].

Previous studies on insect control in low-oxygen treatment have focused on CO_2_ instead of N_2_ [20]. However, there have been concerns about using CO_2_ because it is a greenhouse gas and causes global warming [21]. Moreover, CO_2_ can react with water to produce carbonic acid depending on temperature (generally at 25 °C), while nitrogen does not [22]. In addition, high levels of CO_2_ can also damage fruit quality, including appearance (browning, impair the skin) and the nutritional value (enhance fruit ripening and off-flavor) [23,24]. In postharvest treatment, when using a low-oxygen method with elevated N_2_ to control insects, the fruit quality is acceptable [25]. Therefore, more and more attention has been paid to using N_2_ to replace CO_2_ in the low-oxygen treatment [26]. The high-nitrogen and low-oxygen atmosphere have proven to be environmentally friendly, safe for fruit quality and employees and an effective method for treating insect pests [27]. Moreover, the high-nitrogen and low-oxygen treatments do not reduce the efficacy of some tephritid fruit flies phytosanitary methods such as Irradiation [28,29]. Many studies confirmed that there are no anaerobic with a controlled atmosphere. It has less decay in stored fruit quality at room temperature with low O_2_ (<1%) levels compared to those at average O_2_ (20.9) because a 1% O_2_ level inhibits the growth of most bacteria and molds which cause anaerobic respiration [30].

The atmosphere associated with anaerobic paths due to low-oxygen has a significant role in the occurrence of anaerobic respiration. Nishizawa et al. [25] confirmed that fruit decay did not occur under anaerobic N_2_ atmospheres and low oxygen concentration. Furthermore, anaerobic nitrogen atmospheres resulted in the inhibition of the depolymerization of polyuronides and non-cellulosic neutral sugars in the cell walls, high flesh firmness and low ethylene production when they exposed “Andesu” netted melon fruit to low oxygen concentration with pure nitrogen for 12 days.

Here, we examined low-oxygen/high-nitrogen treatments on *C. capitata* eggs and larvae on a carrot diet, to understand its responses to low oxygen. An artificial diet was chosen, as different fruits vary in composition, nutrients and chemicals, which can influence fruit fly development, life span, longevity and survival; consequently, the difference of fruits will interference with treatments and affect the results. Wang et al. (2018) mentioned many studies in the introduction of his published work [31]. Therefore, the use of an artificial diet minimizes these risks and allows for the focus on fly mortalities in the low-oxygen environment.

## 2. Materials and Methods

### 2.1. Insect Culture

*Ceratitis capitata* used in this experiment was established in 2015 from a laboratory colony preserved at the Department of Primary Industries and Regional Development (DPIRD) in Western Australia. The colony has been periodically supplemented with the introduction of wild flies, and the blending rate was 50:50%. It was reared in the National Centre for Postharvest Disinfestation Research on Mediterranean Fruit Fly at Murdoch University (Perth, Western Australia). The flies were reared in a temperature-controlled cabinet at 23.0 ± 1.0 °C, 60–65% RH and a darkness–light cycle of 12:12 hrs (D:N).

For the artificial diet, 1 kg dehydrated dried carrot (Spices Australia-Herbs & Spices, Dehydrated Food-Ballina, NSW-Australia) was soaked in 5.0 L of hot water for 15 min and homogenised with 330 g Torula Yeast (Lotus-organic foods store- Perth, Western Australia), 30 g methyl propyl hydroxybenzoate (Nipagin) and 33 mL hydrochloric acid (32%, *w*/*w*). Approximately 20 kg of the artificial diet was stored in 4 × 5 L containers (10 cm × 20 cm × 25 cm) [32].

The eggs were then placed on the diet in a plastic tray (20 × 15 × 3 cm^3^), which was moved to a vermiculite-filled plastic tray with 1 cm depth of medium vermiculite size at the bottom), covered with mesh cloth and transferred to an incubator at 25.0 ± 1.0 °C; 70–75% RH until the larvae start to develop into pupae. By sieving the vermiculite, pupae were collected and transferred to screen cages (30 cm^3^ in size) until they developed to adults. Adults were fed with crystalline sugar (Bidvest, Australia), yeast hydrolysate (Australian Biosearch) and water. Each substance was placed in a container separately. The adult females laid their eggs on the sides of the screen cages and those that fell into the water tray adjacent to the cage were collected each day for preparing *C. capitata* required for treatments. To prepare 1st, 2nd and 3rd larval instars, different groups of eggs were placed on a carrot diet and incubated at 25.0 ± 1.0 °C; 70–75% RH for different periods (three days for 1st, five days for 2nd and eight days for 3rd instar). For greater accuracy in the preparation of the larvae, after incubation, a magnifying glass (5×) was used to check the morphological characteristics of each larva to confirm its instar [33]. After verification of each instar, the required number was picked by 5 mL plastic dropper and counted using a manual cell counter.

### 2.2. Low-Oxygen Treatment Facility

**Figure 1:**
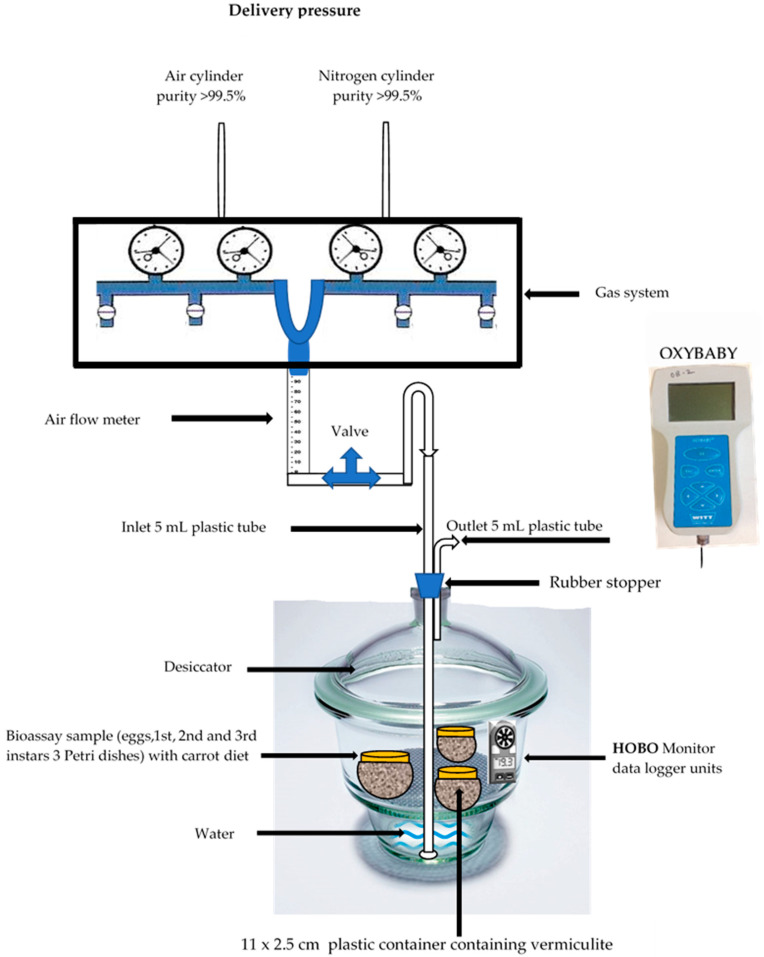
Schematics of low-oxygen treatment experimental setup.

The experimental equipment was installed in a laboratory (25.0 ± 1.0 °C; 35–45% RH) at Murdoch University, Murdoch, WA 6150, Australia. A 2-L glass desiccator (Sigma-Aldrich, Missouri, USA) was used for the low-oxygen treatment of *C. capitata* eggs and different instars. The total volume of the desiccator with lid was 2.610 L. Through the rubber stopper, a plastic tube (5 mm ID clear vinyl non-toxic PVC tubing–5 m. I/N: 3130558, conforming to AS/NZS 2070:1999 for food contact applications) was inserted into the bottom of the desiccator (Figure 1). The air and nitrogen (cylinders G Size, Brand BOC, Industrial Grade, purity >99.5%) flowed through regulators, and tubes to a gas system device (Shimadzu GC-9A, American subsidiary). The device has gauges, nipples and airflow meters to mix, adjust and monitor the gas concentrations. The desired concentrations (0.5% or 5.0%, *v*/*v*) of oxygen and nitrogen (99.5% *v*/*v*) (mixture) were adjusted by the nipples. The gas mixture (O_2_+N_2_) exited from the device by one tube to the desiccator, where it was bubbled through 500 mL distilled water (Figure 1).

Once the desiccator was saturated with the desired O_2_ concentration (0.5% or 5.0%), the gas flow was maintained for the duration of the experiment and flowed through an outlet port. A Witt OXYBABY^®^ 6.0 (WIT-Gasetechnik GmbH and Co KG T, Germany) was used to monitor levels of oxygen and nitrogen (gas mixture) in the desiccators which were placed in an incubator. The O_2_ and N_2_ levels were monitored daily three times with the OXYBABY to avoid any changes in the Shimadzu GC-9A device. The distilled water was added to the desiccator to maintain the humidity inside the desiccator. The volume (500 mL) of distilled water was already calibrated with the gas mixture flow rates, desiccator volume and exposure time. When a monitoring value was observed to have changed from the desired system setup, it was immediately rectified back accordingly. The temperatures and relative humidities inside of desiccator were recorded every 30 min by HOBO® data logger units (Model number H08-004-02, Onset Computer Corporation, MA 02532, USA, www.onsetcomp.com). The HOBO® units had previously been calibrated against a standardised mercury glass thermometer for temperature, and with a range of glycerol/water solutions for relative humidity.

### 2.3. Low-Oxygen Treatment

The prepared diet (55 g) was placed in sterile 9 cm × 10 mm plastic Petri dishes. Eggs were collected from the medfly colony using a plastic pipette. One hundred 24-h-old eggs were placed on the diet surface in each Petri dish. For each low-oxygen treatment (0.5% and 5.0%), a total of 27 Petri dishes were prepared, including triplicates for the nine days of exposure time along with triplicate for day 0 (control group), the total number of Petri dishes are shown in Table S1. Each prepared petri dish was transferred to a plastic container filled with vermiculite (11 cm diameter and 2.5 cm depth), and the container, in turn, was covered with a piece of cloth (mesh) and fixed with a rubber band to prevent the escape of larvae. The three replicate plastic containers containing eggs and 1st, 2nd and 3rd instar larvae were placed into each 2-L treatment desiccator (9 desiccators for each treatment and 1 for control) and transferred into the incubator (model HWS, LET code 0574-88000432, Tianjin- China) set at 25.0 ± 1.0 °C and 70–75% RH. The low-oxygen treatments (0.5% and 5.0%) were applied to each stage of *C*. *capitata* and each replicate included 100 individuals of each insect stage. Gas cylinders placed in the laboratory controlled by an air conditioner, which has an auto sensor set up at 25.0 °C (Acson, AWM-GWRCM-DW/EW ACK-AW/CW ACC-CW ADB-BW, ISO 5151, the temperature setting range is from 16 °C to 30 °C, Malaysia). The time required to obtain the desired oxygen concentration from the gas mixture (O_2_+N_2_) into the desiccators was four hours at a flow rate of 0.6 cubic foot/hour. The gases from the N2 and air cylinders were released into the gas system (Shimadzu GC-9A) then to the desiccators, as shown in Figure 1. The O2 and N2 concentrations in control were 20.9% and 78.09%, respectively, which were regulated at constant rates using the same methods and instruments. One difference between the treatment desiccators and the control desiccator was that an air cylinder was joined to the gas system to maintain the control samples with air only. After the required O_2_ concentrations (0.5% or 5.0%) and planned exposure times were achieved, the Petri dishes were removed from desiccators and placed in the incubator at ambient environmental conditions until they became pupae. The pupae were counted and transferred to sterile 9 mm × 50 mm plastic Petri dishes until they emerged as adults, which were counted and sexed. A total of 24,000 eggs and larvae for 0.5% O_2_ and 5.0% O_2_ (3000 for each stage of egg and 1st, 2nd and 3rd instar) were used through the 0-9 days of exposure to low-oxygen treatment Table S1.

### 2.4. Statistical Analysis

Pupariation and adult emergence were used as criterias on alive individual after exposure to the that low O2 concentrations. Pupariation and emerged adult ratios (%) were utilised to calculate the lethal time (LT). If a treated fly, egg or larva successfully developed into a pupa or an adult, it was considered a survived fly. If not, it was counted as a dead fly. Pupariation and adult emergence rates from control flies were used to normalize the pupariation and adult emergence rate from treated flies according to Schneider-Orelli’s formula [34].

The mortality rate of the insect under low-oxygen treatment was statistically estimated following the lethal time method (LT). Some data of samples were not subject to the normal distribution; therefore, two different models were used on the low-oxygen treatment of eggs and 1st instar, 2nd instar and 3rd instar larvae. They are the probit model on log-transformed treatment days and (Equation (1)) the logit model on log-transformed treatment days. The LT value was (50, 90 and 99%) estimated under a ;generalized linear model with both probit and a logit link function on low-oxygen treatment days. The model is written as:

η = β0 + β1 × χ(1)

where η is the response or proportion mortality, β1 is the coefficient of the dose, β0 is the intercept and χ is the dose. The best model was selected based on the chi-square value (Table S2). The smaller the values of chi-square, the smaller the values of residual will be (residual is the difference between observed values and expected values). The 50, 90 and 99% mortality (LT 50, 90 and 99) were estimated by using the selected models. A 95% confidence interval was reported. Statistical Package for the Social Sciences (SPSS, IBM version 24 Armonk, New York, NY, America) was used to analyse the dose–response data. Experiments were analysed using one-way analysis of variance (ANOVA), followed by Tukey’s honestly significant difference (HSD). The data of sex ratio was transformed to percentage by applying Arcsin square root law prior analysis. The Arcsin law is written as:

(2) $$Y\;=\;\sin^{-1}\;\sqrt{p}$$

where p is the proportion and Y is the result of the transformation.

## 3. Results

**Figure 2:**
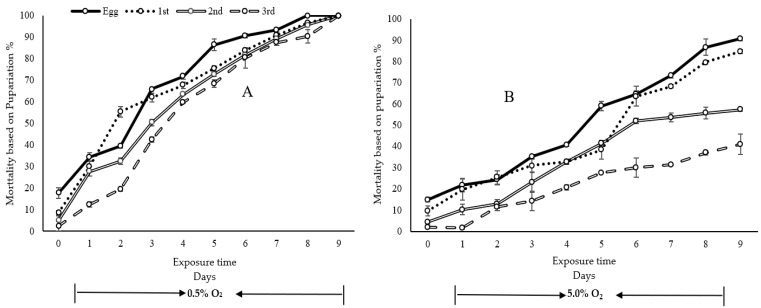
Mortality (SE) of eggs and 1st, 2nd and 3rd stage larvae based on pupariation rate. (**A**)—0.5% O_2_ and (**B**)—5.0% O_2._

The nine-day treatment on larvae (including 1st, 2nd and 3rd instars) resulted in no pupae. However, nine-day treatment under 5.0% O_2_ resulted in 9.3, 15.3, 42.6 and 59.0% pupariation ratios for eggs and 1st, 2nd and 3rd instar larvae, respectively (Figure 2). This result shows that 0.5% O_2_ treatment at 25.0 °C for nine days offers complete control of all studied immature stages of medfly (Figure 2). The pupariation rates decreased with increasing exposure time at low O_2_ treatment. After the treatment, the highest pupariation rate was always recorded from the 3rd instar larvae while the lowest was from the eggs (Figure 2), indicating that the 3rd instar was the most tolerant stage while the egg was the most susceptible stage at the low-oxygen level tested in this study.

**Figure 3:**
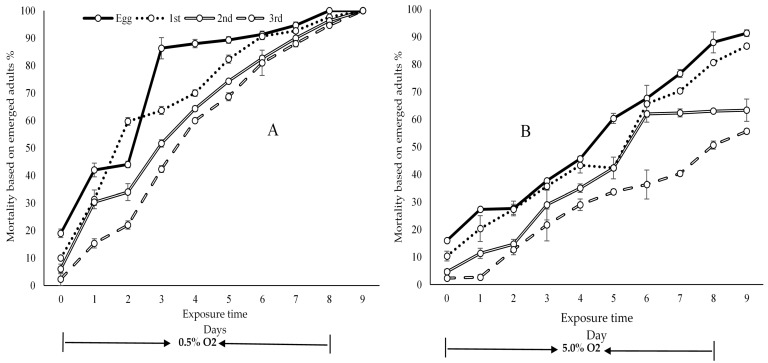
Mortality (SE) of eggs and 1st, 2nd and 3rd stage larvae based on emerged flies rate. (**A**)—0.5% O_2_ and (**B**)—5.0% O_2._

When using pupariation as the endpoint for mortality analysis, the probit model was a better model for eggs and 1st, 2nd and 3rd instar under 0.5% O_2_ treatments and eggs and 2nd and 3rd instar under 5.0% O_2_ treatments where the values of the chi-square with probit model were less than in the logit model (Table S2). For 1st instar larvae under 5.0% O_2_ treatments, the logit model was the better model. The modeled results demonstrate that the 3rd instar was the most tolerant stage with LT_50_, _90_ and _99_ values being 3.8, 7.0 and 10.4 days, respectively, under 0.5% O_2_ treatment, while 9.5, 16.8 and 22.8 days under 5.0% O_2_ treatment (Table 1) were F = 397.94, *p* = 0.00 (Table S3). Similar results were observed in the bioassay that the 3rd instar mortality was always the lowest in both O_2_ concentrations and during all exposure times (Table 1). The 2nd instar was the second most tolerant stage. Eggs were more susceptible (LT_99_ = 8.6) than larvae and their mortality was the highest (Table 1). The results of the emerged adults followed the same pattern as the pupariation, but at a lower rate, as is evident in Figure 3.

When using adult emergence as the endpoint for mortality analysis, the logit model was a better model for eggs and 1st and 3rd instar under 0.5% O_2_ treatments, while the probit model was a better model than logit for the 2nd instar stage, where F = 397.94 and *p* = 0.00 (Table S3). The probit model was a better model for all four stages under 5.0% O_2_ treatment (Table S2). The modelled results revealed that, under 0.5% O_2_ treatments, LT_99_ was 8.5, 8.8, 9.5 and 9.6 days for eggs, 1st instar, 2nd instar and 3rd instar, respectively (Table 1), further confirming that the 3rd instar was the most low-oxygen-tolerant stage (LT_99_ = 9.6) (Table 1). The second is the 2nd instar (LT_99_=9.5) (Table 1), while the 1st instar was the third (LT_99_ = 8.8) (Table 1). The egg was the most susceptible stage to low-oxygen treatment (LT_99_ = 8.5 and 14.3, respectively) (Table 1), where the F value of stages is 397.94 and *p* = 0.00 (Table S3).

### Sex Ratios

**Figure 4:**
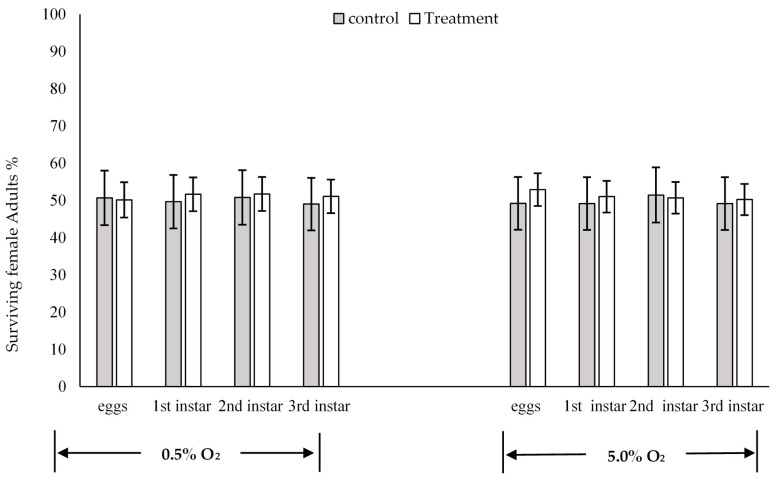
Percentage of surviving adult females under 0.5% O_2,_ and 5.0% O_2_. Error bars represent the standard error of the mean. An ANOVA single factor test was used to compare sex ratios, *p* > 0.05.

There are no significant differences in the female adult ratios among the four stages, which were all close to 50% (Figure 4). To examine whether a certain period of days of treatment influences females survival more than males or opposite, we analysed the female adult ratios from treated flies for 0, 2, 3 or 4 days (Figure S1). The data after Day 4 was not included in the analysis because the numbers of surviving adults are very low (<10). From Day 0 to Day 4, based on the ANOVA test, there are no significant differences between days for the four stages, and the female adult ratios were still close to 50% (Figure S1). Overall, there were no significant effects of low-oxygen treatment on the sex ratio of surviving *C. capitata* in all treatments (insect stages, exposure times and O_2_ concentrations) (Figure 4 and Table S1).

## 4. Discussion

In this work, we chose to test a low-oxygen treatment at high-nitrogen atmosphere to *C. capitata* in the lab diet but not fruits, to avoid host influence on fly physiology and development, resulting in significant differences in fly response. Of the two oxygen treatments, 0.5% O_2_ was more effective than 5.0% O_2_ at killing *C. capitata* eggs and larvae, F = 2895.94 and *p* = 0.00 (Table S3). This result is consistent with Maekawa and Elert [35], who found that there was no survival of *Anthrenus flavipes* (LeConte) under 0.3% oxygen over seven days at 25.6 °C and 33–75% RH while control insects developed naturally.

Exposure time is one of the significant factors in a controlled atmosphere. We designed the exposure time for nine days according to our preliminary data, where nine days under 0.5% O_2_ were enough to kill all stages (eggs and larvae stages) of *C. capitata* on an artificial diet at 25.0 ± 1 °C. Our experimental results show that the number of surviving flies decreased when continually exposed to the 0.5% O_2_ treatment. There were significant effects of exposure days to low-oxygen treatment on medfly, F = 1236.92 and *p* = 0.00 (Table S3). This finding was consistent with earlier studies that refer to increased mortality of flies after they were exposed to low-oxygen concentrations [36]. Yan et al. [37] reported that the number of *Callosobruchus maculatus* larvae that developed to pupae and the number of pupae that emerged to adults was significantly reduced when the exposure time to low concentrations of oxygen (5.0%) was increased compared to control samples.

In the present study, the 3rd instar was found to be the most tolerant of low-oxygen. However, Yahia and Zaleta [38] found that eggs of *Anastrepha obliqua* were more tolerant than the 3rd instar under low-oxygen treatment at 49.4 °C and 54.8 °C with 55 % RH for 220–240 min. In contrast, in the current findings, eggs were more susceptible to low-oxygen treatments (0.5% and 5.0%) than larvae. These results agree with those of Shellie [39] who found that the third instar of the Mexican fruit fly (*Anastrepha ludens*), which was artificially infested into grapefruit, was more tolerant than other stages when exposed to 1% O_2_ and 50% CO_2_ at room temperature. All these results suggest that more attention should be placed on the third instar stage of fruit fly during low-oxygen treatment as it is the most tolerant stage. It is likely that there are many inter-related reasons for the increased survival of the larvae compared to the eggs such as ecological, morphological, physiological and behavioral [40,41,42].

Low-oxygen atmosphere leads to anaerobic metabolism, which results in the production of lactic acid and CO_2_. To release CO_2_ from insect tissues, the insect tracheae spiracles would open fully and rapidly. Thus, faulty spiracular control leads to rapid death due to dehydration [43,44].

The evolution of sex ratio is a complex field of evolutionary ecology. In some cases, environmental factors directly regulate the sex ratio. The evolution of sex ratio may be constrained, e.g., by genetic determination of sex [45]. Sex ratios indicate both the relative survival of females and males and the future breeding potential of a population [46]. Walder et al. [47] observed a difference in sex ratio survival, with mortality rates of females markedly higher than males when they studied the effects of gamma radiation on the sterility and behavioral quality of the Caribbean fruit fly. Some new control strategies of insect pests focus on using methods that affect the sex ratio; for example, environmental effects can create a prejudiced sex ratio by excess production of the sex that is easy to produce under poor environmental conditions [48].

The sex ratios of the emerged adult flies were examined to investigate differences of the male and female larvae mortalities under low-oxygen treatment. There was no significant difference in the sex ratios of flies under low-oxygen treatments at different stages, oxygen concentrations and exposure times (F = 2.08, *p* = 0.10) (Table S3). Moffitt and Albano [49] found that exposure to low oxygen changed the sex ratios of codling moth (*Cydia pomonella*) with more females than males. However, in the present study, we observed that there were slightly more female flies in all oxygen treatments than males, but no significant differences were detected.

This article was a preliminary study to examine medfly responses to low oxygen plus high nitrogen; more work is required before it can be used as a practical application to control flies in fresh produce.

This research will help us to develop protocols that use combinations of lower-dose stresses (low oxygen, irradiation, or cold), which could achieve both higher pest mortality and lower any qualitative impact on the fruits in the future. Nine days are long to control fruit flies in fresh produce, and it is difficult to maintain fruits at a good quality. However, this low-oxygen control method can be considered in combination with other treatment stressors (e.g., low temperature, irradiation, etc.) to make the whole treatment shorter and more efficient. This preliminary study provides knowledge to proceed to develop new treatment methods combined with different stresses.

Furthermore, this study established a lab-based low-oxygen treatment bioassay for the medfly. Based on this method, it is possible to study the molecular basis of medfly responses to low-oxygen treatment at specific treatment times. Compared to flies treated inside fruits, this bioassay, using the naked fly in the diet, removed the impacts of the fruits and helps collect flies quickly and easily. If the treated medfly samples are collected from fruits, the time taken to process the flies is much longer, and the risk of contamination is increased together with the degradation of the samples, particularly RNA samples.

## 5. Conclusions

This is the first experiment to use low oxygen combined with high nitrogen to control the medfly, *C. capitata*, on a carrot diet to be the fundamental database of a low-oxygen and high-nitrogen regime. The third instar larvae were the most tolerant of the low-oxygen treatment. There are no significant differences in the sex ratios of the treated medflies and control flies. Lastly, a laboratory low-oxygen system was established to study the fruit fly under low-oxygen treatments, which can be utilized in further studies, for example, fruit flies in various fruits.

## Figures and Tables

**Table 1:** Low-oxygen treatment duration to induce 50, 90 and 99% mortality based on pupariation and emerged adults from four developmental stages of *C. capitata*.

Treatment	Stage	Mortality Based on Pupariation			Mortality Based on Emerged Adults		
		LT 50	LT 90	LT 99	LT 50	LT 90	LT 99
		(95% *CL)	(95% CL)	(95% CL)	(95% CL)	(95% CL)	(95% CL)
**0.5% O_2_**	Egg	2.8 (2.6–3.1)	6.0 (5.6–6.4)	8.6 (8.0–9.3)	2.1 (1.3–2.8)	5.2 (4.3–6.6)	8.5 (6.9–11.5)
	1st	2.8 (2.3–3.3)	6.5 (5.9–7.4)	9.5 (8.5–11.1)	2.6 (2.1–3.0)	6.0 (5.4–6.8)	8.8 (7.8–10.2)
	2nd	3.2 (2.9–3.4)	6.7 (6.3–7.1)	9.5 (8.9–10.3)	3.3 (3.0–3.5)	6.7 (6.3–7.1)	9.5 (8.9–10.2)
	3rd	3.8 (3.5–4.0)	7.0 (6.6–7.4)	10.4 (9.7–11.3)	3.7 (3.5–4.0)	6.9 (6.6–7.4)	9.6 (9.0–10.2)
**5.0% O_2_**	Egg	5.2 (4.6–5.7)	9.5 (8.5–10.9)	14.1 (12.4–16.8)	4.9 (4.5–5.2)	9.4 (8.8–10.1)	14.3 (13.2–15.7)
	1st	5.6 (5.3–6.0)	10.4 (9.7–11.3)	15.6 (14.4–17.3)	5.3 (5.0–5.6)	10.2 (9.5–11.0)	15.2 (14.2–17.1)
	2nd	6.8 (6.3–7.4)	13.1 (11.9–14.7)	18.2 (16.4–20.7)	6.0 (5.3–6.9)	11.8 (10.3–14.3)	16.6 (14.1–20.6)
	3rd	9.5 (8.6–10.7)	16.8 (14.8–19.7)	22.8 (19.8–27.1)	7.7 (7.2–8.4)	13.9 (12.6–15.7)	19.0 (17.0–21.7)

*CL = confidence limits; LT = lethal time.

## Supplementary Materials

The following are available online at https://www.mdpi.com/2075-4450/11/11/802/s1, Table S1: Experimental design for the control of a medfly on a carrot diet at different O_2_ concentrations and different exposure times at 25 °C ±1 with 70–75% RH. Table S2: Two different models were used in the C. capitate mortality data analysis. Table S3: ANOVA table of low-oxygen concentrations, exposure times, stages and sex ratio of Ceratitis. Capitata. Figure S1: The female percentage (%) of survived adults during the first four days of 0.5% and 5.0% low-oxygen treatments. Error bar means standard error. An ANOVA single factor test was used to compare sex ratios, *p* > 0.05.
