# Supplementary material for: Mediterranean Fruit Fly Ceratitis capitata (Diptera: Tephritidae) Eggs and Larvae Responses to a Low-Oxygen/High-Nitrogen Atmosphere
Source: Insects. 2020 Nov 13;11(11):802. doi: 10.3390/insects11110802 (PMC7696186; doi:10.3390/insects11110802)
Supplement: Supplementary file 1 [file insects-11-00802-s001.zip › Supplementray data.docx]

**Table S1**. Experimental design for control of Medfly on carrot diet at different O_2_ concentrations and different exposure time at 25 ºC ±1 with 70 -75% RH.

| Oxygen con. | Insect developmental stage | | Insect numbers | Number of replicates | Exposure time | Total number of stages in each test |
| --- | --- | --- | --- | --- | --- | --- |
| 0.5% | Eggs | 100 | | 3 | 0-9 days | 3000 |
|  | 1^st^ instar | 100 | | 3 | 0-9 days | 3000 |
|  | 2^nd^ instar | 100 | | 3 | 0-9 days | 3000 |
|  | 3^rd^ instar | 100 | | 3 | 0-9 days | 3000 |
| 5.0% | Eggs | 100 | | 3 | 0-9 days | 3000 |
|  | 1^st^ instar | 100 | | 3 | 0-9 days | 3000 |
|  | 2^nd^ instar | 100 | | 3 | 0-9 days | 3000 |
|  | 3^rd^ instar | 100 | | 3 | 0-9 days | 3000 |
| Total |  |  | | 24 |  | 24000 |

| **Table S2.** Two different models were used in the *C. capitate* mortality data analysis. | | | | | |
| --- | --- | --- | --- | --- | --- |
| O_2_ concentration | **Stages** | **Name** | **Type** | **Chi-square** | **Method** |
| 0.5% | Eggs | Model 1 | Probit | 14.66 | Pupation |
| 0.5% | Eggs | Model 2 | Logit | 14.89 | Pupation |
| 0.5% | 1^St^ | Model1 | Probit | 17.22 | Pupation |
| 0.5% | 1^st^ | Model 2 | Logit | 18.96 | Pupation |
| 0.5% | 2^nd^ | Model 1 | Probit | 11.38 | Pupation |
| 0.5% | 2^nd^ | Model 2 | Logit | 13.18 | Pupation |
| 0.5% | 3^rd^ | Model 1 | Probit | 14.57 | Pupation |
| 0.5% | 3^rd^ | Model 2 | Logit | 14.49 | Pupation |
| 5% | Eggs | Model 1 | Probit | 21.429 | Pupation |
| 5% | Eggs | Model 2 | Logit | 17.890 | Pupation |
| 5% | 1^St^ | Model1 | Probit | 12.366 | Pupation |
| 5% | 1^st^ | Model 2 | Logit | 10.238 | Pupation |
| 5% | 2^nd^ | Model 1 | Probit | 14.077 | Pupation |
| 5% | 2^nd^ | Model 2 | Logit | 16.190 | Pupation |
| 5% | 3^rd^ | Model 1 | Probit | 12.212 | Pupation |
| 5% | 3^rd^ | Model 2 | Logit | 14.283 | Pupation |
| 0.5% | Eggs | Model 1 | Probit | 43.546 | Adult |
| 0.5% | Eggs | Model 2 | Logit | 39.392 | Adult |
| 0.5% | 1^St^ | Model1 | Probit | 16.079 | Adult |
| 0.5% | 1^st^ | Model 2 | Logit | 16.458 | Adult |
| 0.5% | 2^nd^ | Model 1 | Probit | 8.684 | Adult |
| 0.5% | 2^nd^ | Model 2 | Logit | 10.563 | Adult |
| 0.5% | 3^rd^ | Model 1 | Probit | 12.664 | Adult |
| 0.5% | 3^rd^ | Model 2 | Logit | 12.853 | Adult |
| 5% | Eggs | Model 1 | Probit | 14.292 | Adult |
| 5% | Eggs | Model 2 | Logit | 12.031 | Adult |
| 5% | 1^St^ | Model1 | Probit | 6.987 | Adult |
| 5% | 1^st^ | Model 2 | Logit | 6.465 | Adult |
| 5% | 2^nd^ | Model 1 | Probit | 18.215 | Adult |
| 5% | 2^nd^ | Model 2 | Logit | 19.797 | Adult |
| 5% | 3^rd^ | Model 1 | Probit | 14.195 | Adult |
| 5% | 3^rd^ | Model 2 | Logit | 16.674 | Adult |
| The models including probit and logit. The underlined model was the selected one to calculate LT50, 90, and LT99 in table 1, the best model is the lowest Chi-square value. | | | | | |

| Source | Type III Sum of Squares | df | Mean Square | F | Sig. |
| --- | --- | --- | --- | --- | --- |
| Corrected Model | 222703.600 | 79 | 2819.033 | 207.345 | .000 |
| Intercept | 622609.067 | 1 | 622609.067 | 45794.108 | .000 |
| Stages | 16231.133 | 3 | 5410.378 | 397.944 | .000 |
| Concentrations | 39372.817 | 1 | 39372.817 | 2895.947 | .000 |
| Days | 151353.600 | 9 | 16817.067 | 1236.928 | .000 |
| Sages * Concentrations * Days | 15746.050 | 66 | 238.577 | 17.548 | .000 |
| Error | 2175.333 | 160 | 13.596 |  |  |
| Total | 847488.000 | 240 |  |  |  |
| ANOVA Table of Sex Ratio | | | |  |  |
| Between Groups | 11.722 | 4 | 2.931 | 2.083 | .104 |
| Within Groups | 49.245 | 35 | 1.407 |  |  |
| Total | 60.968 | 39 |  |  |  |

**Table S3.** ANOVA table of low-oxygen concentrations, exposure times, stages and sex ratio of *Ceratitis. Capitata*.


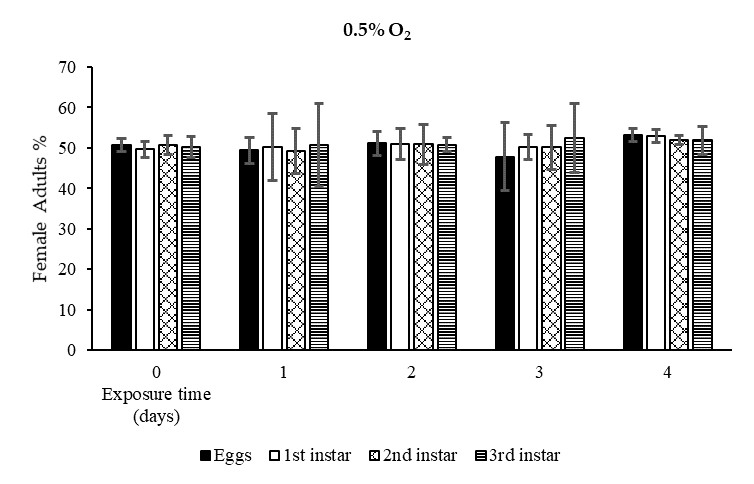

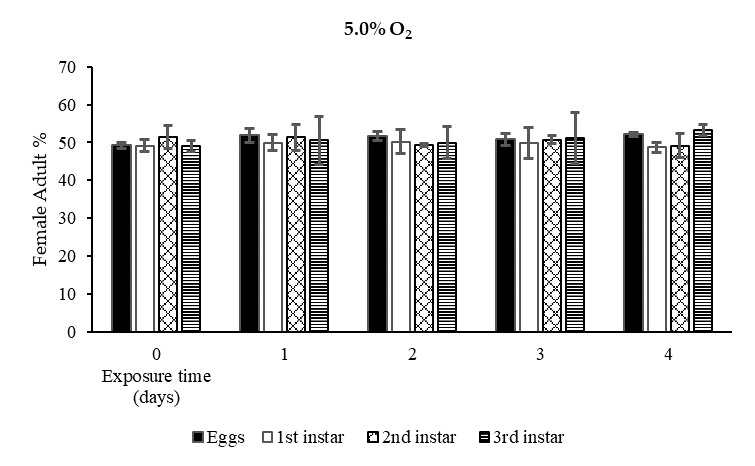


**Figure S1.** The female percentage (%) of survived adults during the first four days of 0.5% and 5.0% low-oxygen treatments. Error bar means standard error. An ANOVA single factor test was used to compare sex ratios, *p* >0.05.
